# Novel Genotype–Phenotype Correlations in *CRB1*-Retinopathies: Insights from Isoforms and Protein Domains Linked to Disease Severity

**DOI:** 10.1016/j.xops.2025.101010

**Published:** 2025-11-15

**Authors:** Ana Catalina Rodriguez-Martinez, Cécile Méjécase, Vijay K. Tailor-Hamblin, Bethany E. Higgins, Robert H. Henderson, Mariya Moosajee

**Affiliations:** 1 University College London Institute of Ophthalmology, London, United Kingdom; 2 Moorfields Eye Hospital National Health Service Foundation Trust, London, United Kingdom; 3 Great Ormond Street Hospital for Children National Health Service Foundation Trust, London, United Kingdom; 4 The Francis Crick Institute, London, United Kingdom; 5 University College London Experimental Psychology, London, United Kingdom

**Keywords:** CRB1, CRB1-retinopathy, Crumbs homolog 1, Genotype–phenotype, Inherited retinal disease

## Abstract

**Objective:**

This study evaluates genotype–phenotype correlations in *CRB1*-retinopathies using standardized phenotypic classification and comprehensive analysis of Crumbs homolog 1 (CRB1)-A and CRB1-B involvement alongside in silico protein modeling analysis.

**Design:**

Retrospective multicenter cohort study.

**Subjects:**

A total of 389 patients with biallelic disease-causing *CRB1* variants from 50 international cohorts, including 73 patients from Moorfields Eye Hospital.

**Methods:**

Phenotypes were reclassified using standardized diagnostic criteria. Genotype–phenotype correlations were assessed based on CRB1 isoform involvement and protein domain localization of variants, supported by in silico structural modeling.

**Main Outcome Measures:**

Associations between *CRB1* variant location, isoform involvement, and clinical phenotypes including Leber congenital amaurosis/early onset severe retinal dystrophy (LCA/EOSRD), retinitis pigmentosa (RP), cone-rod dystrophy, and macular dystrophy (MD).

**Results:**

All patients had variants affecting CRB1-A, with none exclusively affecting CRB1-B. Mutations specific to CRB1-A, sparing CRB1-B were associated with MD. Mutations in exons 6, 7, and 9 were associated to LCA/EOSRD and RP phenotypes, whereas exon 2 variants were linked to MD. Genotype–phenotype correlations included c.1841G>T p.(Gly614Val) linked to LCA/EOSRD and variants exclusively involving exon 11 and 12. Similarly, the variants c.2506C>A p.(Pro836Thr) and c.498_506del p.(Ile167_Gly169del) were linked to MD.

**Conclusions:**

Crumbs homolog 1-A must be affected for disease manifestation, while sparing of CRB1-B leads to milder phenotypes. Novel genotype–phenotype correlations were found using standardized phenotypic classification. Understanding protein structure and isoform involvement is crucial for accurate diagnosis, prognosis, and the development of targeted therapies.

**Financial Disclosure(s):**

The authors have no proprietary or commercial interest in any materials discussed in this article.

The Crumbs (crb) protein, initially identified in the apical membrane of Drosophila epithelial cells,^1^ became relevant when den Hollander et al^2^ discovered that variants in the human homolog, Crumbs homolog 1 (CRB1), cause a diverse spectrum of retinopathies.^3^^,^^4^ Crumbs is part of a 3-member family of CRB1 proteins, alongside CRB2 and CRB3.^5^ The human *CRB1* gene (Online Mendelian Inheritance in Man [OMIM] #604210), located on chromosome 1q31.3, spans over 210 kb and comprises 12 exons, encoding 3 isoforms: CRB1-A, B, and C.^5^ The canonical *CRB1-A* transcript contains 12 exons and is translated into a 1406 amino acid protein.^5^ Its structure includes 19 epidermal growth factor-like repeat domains and 3 laminin-globular-like domains in its extracellular N-terminus, along with a brief FERM/PDZ-binding motif in the intracellular C-terminal domain^6^ and it is primarily expressed in Müller glia cells.^7^ The CRB1-B isoform, comprising 1003 amino acids, shares structural similarity with CRB1-A in the extracellular domains but possesses distinctive 5′ and 3′ domains, and is primarily expressed in photoreceptors.^5^^,^^7^^,^^8^ In contrast, CRB1-C, comprising 754 amino acids, lacks transmembrane and intracellular domains, and its function remains uncertain.^9^^,^^10^

Crumbs homolog 1 acts as a vital regulator of cellular processes, including apical-basal polarity, outer limiting membrane integrity, cell–cell adhesion, and cellular signaling pathways.^5^ It is crucial for retinal development and long-term integrity, particularly in maintaining zonula adherence junctions at the external limiting membrane.^5^^,^^11^ Within the adherence junctions, CRB1 is found in complex with the protein associated with lin seven 1 (PALS1; also known as MPP5), PALS1-associated tight junction protein, and the multi-PDZ domain protein 1.^12^^,^^13^ Together, these proteins regulate signaling pathways and cell fate^14^ by inhibition of the mammalian target of rapamycin complex 1 pathway through interactions between PALS1-associated tight junction and tumor suppressor gene and by modulation of the Hippo pathway, affecting yes-associated protein and transcriptional coactivator with PDZ-binding motif protein activity to control their inhibition and cytoplasmic retention.^15^

Biallelic variants in *CRB1* result in a broad clinical spectrum of autosomal recessive retinopathies.^2^^,^^3^ It has been ranked as the 10th most prevalent molecular cause of inherited retinal diseases in the United Kingdom, accounting for 2.1%.^16^ *CRB1-*retinopathies display distinctive findings including nummular pigmentation, fine yellow punctate deposits, preserved para-arteriolar retinal pigment epithelium, and bone spicules, with some developing a Coats-like vasculopathy (retinal exudation and telangiectasias).^3^ OCT shows a thickened, unlaminated retinal structure with some instances of low grade of foveal hypoplasia (grade 1).^17^^,^^18^ More recently, high intraocular pressure and angle-closure glaucoma has also been associated with *CRB1* retinopathies when harboring the c.2506C>A p.(Pro836Thr) variant.^19^ The most prevalent phenotype is Leber congenital amaurosis (LCA) (OMIM #613935, LCA8) accounting for up to 17% of cases.^18^ Patients with LCA typically present within the first few months of life with poor vision (worse than 20/200 or 6/60), associated with nystagmus, eye poking, poor pupil responses, and in most instances, an undetectable full field electroretinogram (ERG).^3^^,^^20^^,^^21^ Interestingly, although there is no OMIM entry of *CRB1* as a cause of early onset severe retinal dystrophy (EOSRD), this phenotype has been reported in *CRB1*-retinopathies cohorts.^7^^,^^17^^,^^18^^,^^22^ Patients with EOSRD present after infancy and usually before the age of 5 years. Distinguishing features of EOSRD include better baseline vision and milder photoreceptor dysfunction seen on ERG compared with the LCA phenotype.^21^ Because there is significant overlap between the age of onset and symptoms of LCA and EOSRD many intermingle them together as LCA/EOSRD.^21^ Retinitis pigmentosa (RP) is the second most common reported *CRB1*-retinopathy phenotype, accounting for 9% of all RP cases (OMIM #600105, RP12).^22^ Children older than 5 years with later onset signs and symptoms of a retinal dystrophy are termed “juvenile onset” or labeled synonymously as autosomal recessive RP.^3^ Juvenile RP has a disease onset before the age of 20 years, manifesting initially with night blindness, visual field abnormalities, reduced visual acuity and a reduced ERG amplitude (>50%).^3^^,^^23^ Most patients develop a maculopathy and up to 50% experience cystoid macular edema.^24^ Finally, patients with both the cone-rod dystrophy (CRD) and macular dystrophy (MD) phenotypes present in early adulthood, exhibiting initial changes in their central vision, with CRD then having peripheral involvement.^17^^,^^18^^,^^22^^,^^25^, ^26^, ^27^ Within the MD phenotype, observed findings are localized to the macula intriguingly sparing the foveola.^26^ This latter phenotype has an unusual degeneration pattern affecting the superior, inferior, and nasal retina close to the optic nerve, one that is shared with retinopathies caused by *ADAM9* and *CDH3*.^26^

Apart from the established genotype–phenotype correlation of the MD phenotype with the in-frame deletion c.498_506del p.(Ile167_Gly169del),^26^ clear genotype–phenotype correlations in *CRB1*-retinopathies remain limited. This may arise from inconsistencies in phenotypic classification, limited understanding of isoform-specific contributions to disease and CRB1 protein domain structure. This retrospective observational study re-evaluates genotype–phenotype correlations in *CRB1*-retinopathies, applying standardized phenotypic classification and comprehensive analysis of published international cohorts, including Moorfields Eye Hospital, with a focus on the roles of CRB1-A and CRB1-B isoforms and in silico protein structure modeling.

## Methods

A retrospective observational study at a single tertiary referral center (Moorfields Eye Hospital National Health Service Foundation Trust, London, United Kingdom) was performed. Subjects were identified from the prospectively consented Moorfields Eye Hospital Inherited Eye Disease Database (Research Ethics Number: 12/LO/0141), London – Camden and Kings Cross Research Ethics Committee, and all procedures adhered to the tenets of the Declaration of Helsinki. Data for these studies are collected as part of standard of care and retrospectively analyzed. The inclusion criteria was molecularly confirmed biallelic (pathogenic or likely pathogenic) variants in the *CRB1* gene. The methodology of genetic testing and variant interpretation at Moorfields has been described previously.^28^

Demographics, clinical data, past medical and ophthalmic history, refractive error, fundoscopy, retinal imaging, and best-corrected visual acuity (BCVA) were collected from full ophthalmic assessments conducted at each visit as part of their routine clinical care. Best-corrected visual acuity was converted to logarithm of the minimum angle of resolution (LogMAR) for statistical analysis. Count fingers vision was given a value of LogMAR 1.98 and hand motion, LogMAR 2.28; light perception and no light perception were LogMAR 2.7 and 3, respectively.

### Literature Review

A literature review was also conducted in August 2023 using the following databases: Medline via Ovid; Embase, PubMed, and Google Scholar with no date restrictions for articles published in the English language using the following keywords and Boolean operators. The studies were assessed for suitability using a web-based screening software (Covidence, Veritas Health Innovation; available at http://www.covidence.org). If the title and abstract were relevant, an article was reviewed in full. Articles not describing genotype or phenotype description were excluded after review. Search teams included were “CRB1” OR “Crumbs homologue 1,” AND “retinitis pigmentosa” OR “RP” OR “RP12” OR “Juvenile retinitis pigmentosa” OR “Macular dystrophy/ies” OR “MD” OR “retinal dystrophy/ies” OR “early onset severe retinal dystrophy/ies” OR “EOSRD” OR “early onset retinal dystroph∗” OR “EORD” OR “leber congenital amaurosis” OR “LCA” OR “LCA8” OR “cone-rod dystrophy/ies” OR “CRB1-associated.” The study selection process is shown in the Preferred Reporting Items for Systematic reviews and Meta-Analyses (PRISMA) diagram (Supplemental material, available at www.ophthalmologyscience.org). Articles lacking genotype or phenotype descriptions were excluded after review. Variant pathogenicity was validated using databases such as ClinVar, Varsome, and Leiden Open (source) Variation Database. Exclusion of previous papers published from the same institution was done to avoid overlap of cases.^22^^,^^26^

### Classification

Extensive clinical data was collected for this re-evaluation, including demographics, age of onset, visual acuity (BCVA), fundoscopy findings, retinal imaging visual fields, and electrophysiology tests. The re-evaluation and recategorization of phenotypic labels were systematically performed internally by 2 ophthalmologists (A.C.R.-M. and M.M.) with specialized expertise in inherited retinal diseases and ocular genetics. In brief, for each patient, their phenotype was reanalyzed according to the following clinical parameters: (1) patients with LCA/EOSRD present within the first few months of life with poor vision, nystagmus, poor pupil responses, and undetectable full field ERG; or present after infancy, before the age of 5 years, having some residual visual function but significantly reduced (albeit detectable) ERG signals; (2) patients with RP disease onset between 5 and 20 years, complaining of night blindness, visual field abnormalities, reduced visual acuity, and ERG amplitude reduction (>50%)^19^; (3) patients with MD exhibiting initial changes in the quality of their central vision and present at a later age, resulting in a milder manifestation;^29^ and (4) patients with CRD exhibit disturbances in their central vision followed by peripheral vision changes. Increased disease severity was defined as presenting with the LCA/EOSRD ± macular atrophy phenotype, whereas less severe disease was defined by presenting with the MD or CRD phenotype. Although the initial intention was to separate LCA and EOSRD, the retrospective design of the study and the challenges of reliably distinguishing these phenotypes across diverse international cohorts, particularly when relying on historical data and age of onset, made it unfeasible to provide separate prevalence and analyses. To avoid introducing further inconsistencies, unification was decided, which has also been highlighted in the literature, leading to many studies intermingling the 2 groups together as LCA/EOSRD.^21^ This approach was chosen to strengthen the validity of genotype–phenotype correlations by ensuring a consistent definition across all patients.

### Protein Modeling

In silico modeling and prediction of missense variants on protein structure were analyzed with AlphaFold (https://alphafold.ebi.ac.uk; AF-P82279-F1), Missense3D^30^ (http://missense3d.bc.ic.ac.uk/∼missense3d/), and PyMOL Molecular Graphics System (version 2.5.8, Schrödinger, LLC).

### Statistical Analysis

Parametric variables were assessed using linear regressions and *t* tests, whereas analysis of variance was used for multiple comparisons. For nonnormally distributed data, nonparametric tests were used to compare medians. Statistical significance was considered if *P* < 0.05. All statistical analyses were completed using GraphPad Prism (version 8.0.0 GraphPad Software).

## Results

**Figure 1:**
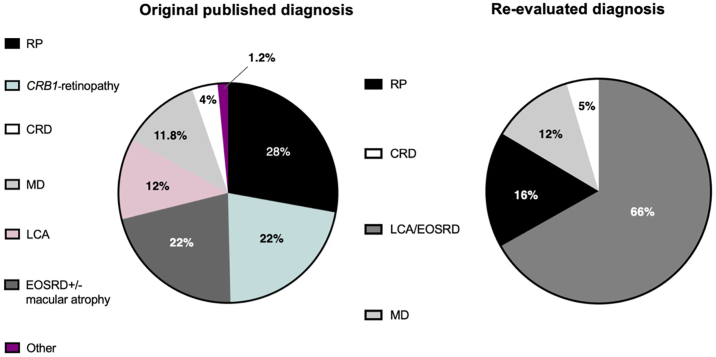
Recategorization of predetermined diagnoses across various cohorts revealed 4 primary phenotypic presentations. After a detailed reassessment of clinical data from each patient, cases were reclassified into Leber congenital amaurosis (LCA)/early onset severe retinal dystrophy (EOSRD), retinitis pigmentosa (RP), macular dystrophy (MD), and cone-rod dystrophy (CRD).

**Figure 2:**
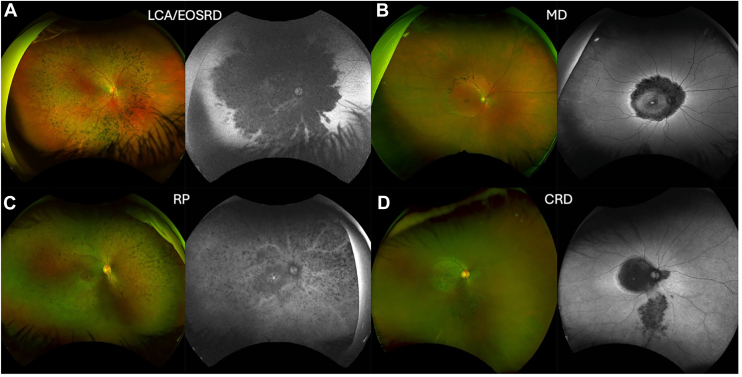
*CRB1*-retinopathies depict a wide spectrum of inherited retinal disease phenotypes. Widefield color fundus photographs and corresponding fundus autofluorescence (FAF) images. **A,** Right eye of a 17-year-old female patient with *CRB1*-Leber congenital amaurosis (LCA8)/early onset severe retinal dystrophy (EOSRD) showing the nummular pigmentation and generalized retinal degeneration seen as hypo-autofluorescence (AF) in the FAF image (**B**). Right eye of a 21-year-old patient with *CRB1*-retinitis pigmentosa (RP) characterized by the para-arteriolar retinal pigment epithelium and bone spicules. **C,** Right eye of a 30-year-old patient with *CRB1*-macular dystrophy (MD) revealing hypo-AF in the posterior pole with a surrounding hyper-AF ring. **D,** Right eye of a 33-year-old patient with *CRB1*-cone-rod dystrophy (CRD) showing pigmentary changes and retinal pigment epithelium atrophy mainly in the posterior pole and inferior retina with corresponding hypo-AF in those regions on the FAF image.

**Figure 3:**
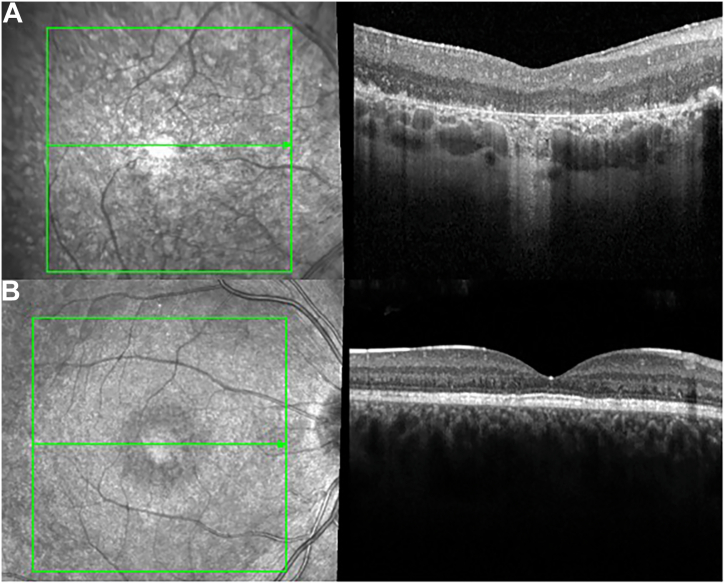
Abnormal retinal architecture seen in spectral-domain OCT imaging is linked to disease severity in *CRB1*-retinopathies. **A,** Spectral-domain OCT imaging of the right eye of a 17-year-old female patient with *CRB1*-Leber congenital amaurosis (LCA)/early onset severe retinal dystrophy (EOSRD) depicting a coarse and thickened retina with retinal disorganization, thinning of the outer nuclear layer (ONL), loss of the ellipsoid zone and foveal hypoplasia grade 1B. **B,** Spectral-domain OCT scan of an 18-year-old male patient with macular dystrophy (MD) displaying ONL thinning, and disruption of the foveal ellipsoid zone yet maintains overall good retinal lamination.

A total of 389 patients with *CRB1*-retinopathy from 325 unrelated families from 49 international published cohorts^7^^,^^17^^,^^19^^,^^24^^,^^27^^,^^31^, ^32^, ^33^, ^34^, ^35^, ^36^, ^37^, ^38^, ^39^, ^40^, ^41^, ^42^, ^43^, ^44^, ^45^, ^46^, ^47^, ^48^, ^49^, ^50^, ^51^, ^52^, ^53^, ^54^, ^55^, ^56^, ^57^, ^58^, ^59^, ^60^, ^61^, ^62^, ^63^, ^64^, ^65^, ^66^, ^67^, ^68^, ^69^, ^70^, ^71^ and Moorfields Eye Hospital (*n* = 73) where genotype and phenotype information was provided were included in the analysis. Among the 389 patients included in this study, 10 predetermined diagnoses were collected across different cohorts including 110 (28.3%) patients categorized as RP, 86 (22.1%) as “*CRB1*-retinopathy” (later reclassified as 79 LCA/EOSRD, 6 RP, and 1 CRD), 81 (20.8%) as EOSRD, 47 (12.1%) as LCA, 46 (11.8%) as MD, 15 (3.9%) as CRD, 4 (1.0%) as EOSRD with macular atrophy, 2 (0.5%) as asymptomatic fenestrated slit maculopathy, 2 (0.5%) as fenestrated sheen MD, and 2 (0.5%) as retinoschisis. After a detailed re-evaluation of each individual’s clinical information, 260 (66%) patients were categorized as LCA/EOSRD, 65 (16%) as RP, 46 (12%) patients as MD, and 18 (5%) as CRD. Excluding the 86 (22.1%) patients initially labeled as “*CRB1*-retinopathy,” 64 (16%) patients were reclassified into different phenotypes groups, with 53 (13%) reclassified as LCA/EOSRD, the majority of whom were originally diagnosed with RP (45 cases, 11%) (Figure 1, Figure 2, Figure 3).

The median age of onset (interquartile range) for LCA/EOSRD was 3 (1, 4) years, 18 years (10.5, 24.5) years for MD, 11 (7, 34) years for CRD, and 13.5 (7, 20) years for RP. The median age of patients (interquartile range) for LCA/EOSRD was 17.5 (6, 29) years, 30 (21, 44) years for MD, 28 (14, 46) years for CRD, and 33 (19, 48) years for RP. Gender distribution was nearly equal (146 females, 145 males; data available for 291 individuals). Ethnic distribution showed predominance of White (38%) and Asian (25%) populations, with smaller representations of Black (4%), Hispanic (4%), and Micronesian (1%) backgrounds. Ethnicity was not specified in 95 individuals (24%).

**Figure 4:**
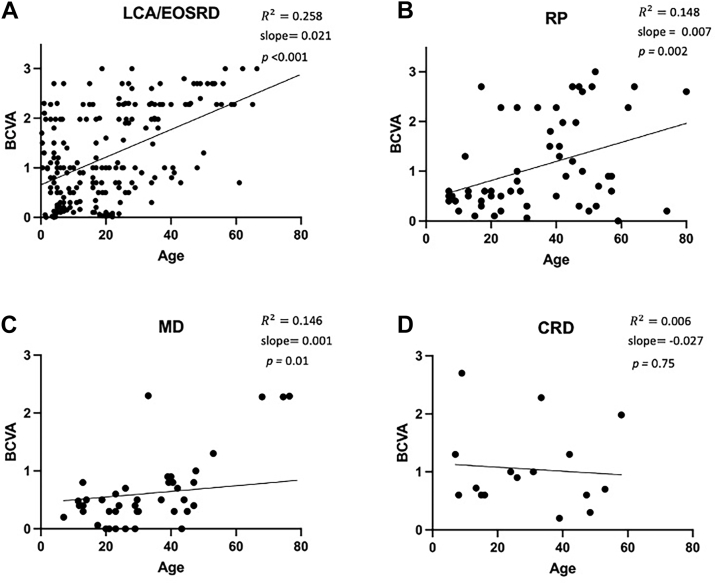
Best-corrected visual acuity (BCVA) age-related decline is more severe in *CRB1* patients with the Leber congenital amaurosis (LCA)/early onset severe retinal dystrophy (EOSRD) phenotype. Scatterplots with linear regression analysis showing the relationship between BCVA and age in *CRB1* patients. **A,** A positive correlation was found between BCVA and age in the LCA/EOSRD phenotype (*n* = 260), with a steep slope (*R*^2^ = 0.258, slope = 0.021, *P* < 0.001). **B,** The macular dystrophy (MD) group (*n* = 46) depicted the most gradual positive slope (*R*^2^ = 0.14, slope = 0.001, *P* = 0.01) **C,** A positive slope was also observed in the retinitis pigmentosa (RP) group (*n* = 65) (*R*^2^ = 0.148, slope = 0.007, *P* = 0.002). **D,** No correlation was observed in the cone-rod dystrophy (CRD) group (*n* = 18) (*R*^2^ = 0.006, slope = –0.027, *P* = 0.75).

The mean BCVA (standard deviation) for the groups were as follows: LCA/EOSRD 1.22 LogMAR (± 0.91) (confidence interval [CI]: 1.107–1.35), MD 0.63 LogMAR (± 0.61) (CI: 0.44–0.82), RP 1.06 LogMAR (± 0.90) (CI: 0.83–1.30), and CRD 0.94 LogMAR (± 0.73) (CI: 0.58–1.31). Simple linear regression showed a positive correlation between BCVA and age, indicating that older age is associated with worsening of vision, noticing a steeper line for LCA/EOSRD (*R*^2^ = 0.258, slope = 0.021*, P* ≤ 0.001), followed by RP (*R*^2^ = 0.148, slope = 0.007*, P* = 0.002) and MD (*R*^2^ = 0.14, slope = 0.001*, P* = 0.01) phenotypes, but with no correlation in the CRD group (*R*^2^ = 0.006, slope = –0.027*, P* = 0.75) (Fig 4).

### Molecular Characteristics

**Figure 5:**
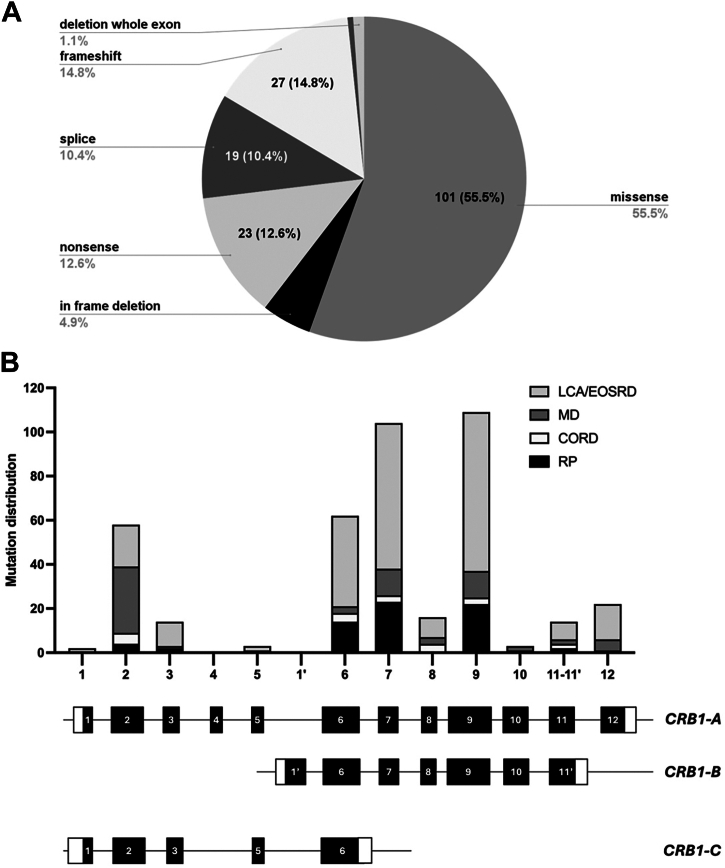
All patients had biallelic variants affecting CRB1-A, with no cases exhibiting mutations exclusively in CRB1-B or CRB1-C. Types of mutations reported in patients with *CRB1*-retinopathy and their distribution across CRB1-A and CRB1-B. **A,** The most common type of mutation observed in *CRB1*-retinopathies was missense, followed by frameshift mutations and nonsense mutations. **B,** Distribution of mutations across the 12 exons of the canonical transcript encoding CRB1-A and the corresponding exons comprising the shorter CRB1-B transcript and CRB1-C transcript. Hotspot mutations were identified in exons 6, 7, and 9, primarily associated with Leber congenital amaurosis (LCA)/early onset severe retinal dystrophy (EOSRD) and retinitis pigmentosa (RP), whereas exon 2 contained hotspot mutation associated with macular dystrophy (MD). No mutations were identified that exclusively affected CRB1-B, including those within the 1′ region. Variants impacting the 11′ region, which is specific to the CRB1-B transcript, also affected CRB1-A as splice variants. Similarly, no variants were found that exclusively affected CRB1-C, including those within the 6′ region. CORD = cone-rod dystrophy; CRB1 = Crumbs homolog 1.

One hundred ninety-six unique likely pathogenic or pathogenic variants were reported in patients with *CRB1*-retinopathies included in this study (Fig 5): 101 were missense (55%), 27 frameshift (14.8%), 23 (12.6%) nonsense, 19 (10.4%) splice, 9 (4.9%) in-frame deletion; and <1% included exon deletion and in-frame insertion (Fig 5A). Of the 46 patients who had either frameshift mutations, nonsense mutations, or abnormal splicing, 43 (93%) were diagnosed as LCA/EOSRD, whereas 3 (6%) had RP. Variants associated with *CRB1*-RP phenotype were mostly located in exon 6 (*n* = 14), exon 7 (*n* = 23), and exon 9 (*n* = 22) (Fig 5B). Similarly, variants causing LCA/EOSRD were frequently localized in exon 6 (*n* = 41), exon 7 (*n* = 66), and exon 9 (*n* = 72), both of which affect isoforms CRB1-A and CRB1-B (Fig 5B). Variants associated with MD were mainly located in exon 2 (*n* = 40). Variants associated with CRD span all the exons.

Figure 5B depicts the distribution of variants across the 3 *CRB1* isoforms: A, B, and C. The *CRB1-B* transcript contains unique regions, including segment 1′ and 11′. Segment 1′ starts from c.1172–7051 (chr1:1977413949) to c.1172–7000 (chr1:197414000) corresponding to the *CRB1*-*A* transcript (NM_201253.2). Segment 11′ extends from c.4005+1 to +126 (chr1:197442418) of *CRB1-A* transcript (NM_201253.2). Comparably, the *CRB1-C* transcript features a distinct region, 6′, corresponding to c.1009+110 of *CRB1-A* (NM_201253.2). No variants were found that exclusively affect *CRB1-B* or *CRB1*-*C*. Variants affecting segment 11′ of *CRB1-B* also affected *CRB1-A* through splice variants. These variants included c.4005+1G>A and c.4005+2T>G in *CRB1-A*.

**Figure 6:**
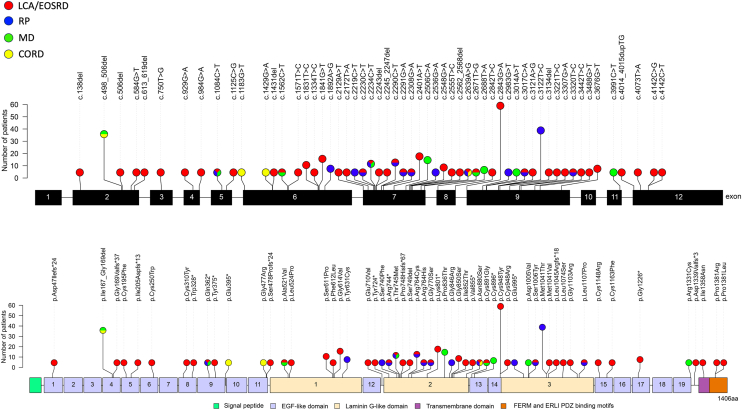
Disease-causing variants found in >1 *CRB1*-retinopathy patient are distributed along *CRB1-A* (NM_201253.2) and the corresponding CRB1-A protein structure (NP_957705.1). Signal peptide (amino acid position 1–25) depicted in green, extracellular domain (amino acid position 26–1347) depicted in lilac and yellow; transmembrane domain (amino acid position 1348–1368) depicted in purple; and cytoplasmatic domain (amino acid position 1369–1406) depicted in orange. Nonsense, frameshift, in-frame deletion, and missense variants are reported; the associated phenotype is color-coded: Leber congenital amaurosis (LCA)/early onset severe retinal dystrophy (EOSRD) is depicted in red, retinitis pigmentosa (RP) in blue, macular dystrophy (MD) in green, and cone-rod dystrophy (CORD) in yellow and the number of patients carrying the variants are detailed.

The most common variants seen across different cohorts was the missense c.2843G>A p.(Cys948Tyr) located in exon 9, followed by the missense c.3122T>C p.(Met1041Tyr) also located in exon 9; both are associated with LCA/EOSRD and RP phenotypes and both seen more frequently in White patients (Figure 5, Figure 6 and Table 1). This was followed by the in-frame deletion c.498_506del p.(Ile167_Gly169del), which was associated with the MD phenotype and CRD to a lesser degree, commonly seen in White and mixed/other populations. The most common variants seen in Asian cohorts were the c.1831T>C p.(Ser611Pro) and c.1841G>T p.(Gly614Val), the latter strongly correlated LCA/EOSRD as both homozygous and compound heterozygous. Intrafamilial phenotypic variation was rare in this study. Among the 40 families with >1 affected family member, phenotypic heterogeneity was observed in only 4 families, who were homozygous for c.2639A>G p.(Asn880Ser) or c.3122T>C p.(Met1041Thr) variants.

**Table 1:** The 10 Most Frequent *CRB1* Variants Found Across Cohorts

Variant Number	cDNA Change	Protein Change	Type of Mutation	Exon	Phenotype	Frequency %	CRB1-B Affected
1	c.2843G>A	p.(Cys948Tyr)	Missense	9	Overall	16.97%	Yes
					• LCA/EOSRD	71.2%	
					• RP	13.6%	
					• MD∗	15.2%	
2	c.3122T>C	p.(Met1041Thr)	Missense	9	Overall	10.80%	Yes
					• LCA/EOSRD	71.4%	
					• RP	26.2%	
					• CRD	2.4%	
3	c.498_506del	p.(Ile167_Gly169del)	In-frame deletion	2	Overall	10.03%	No
					• MD	87.2%	
					• CRD	12.8%	
4	c.2401A>T	p.(Lys801∗)	Nonsense	7	Overall	7.20%	Yes
					• LCA/EORD	75%	
					• MD∗	21.4%	
					• CRD	3.6%	
5	c.1841G>T	p.(Gly614Val)	Missense	6	Overall	8.23%	Yes
					• LCA/EORD	100%	
6	c.2506C>A	p.(Pro836Thr)	Missense	7	Overall	4.11%	Yes
					• MD	43.8%	
					• RP	43.8%	
					• CRD	12.5%	
7	c.2290C>T	p.(Arg764Cys)	Missense	7	Overall	4.37%	Yes
					• LCA/EORD	64.7%	
					• RP	35.5%	
8	c.2234C>T	p.(Thr745Met)	Missense	7	Overall	3.08%	Yes
					• LCA/EORD	75%	
					• MD	16.7%	
					• RP	8.3%	
9	c.1831T>C	p.(Ser611Pro)	Missense	6	Overall	2.2%	Yes
					• LCA/EORD	92.3%	
					• RP	7.7%	
10	c.2548G>A	p.(Gly850Ser)	Missense	7	Overall	2.57%	Yes
					• LCA/EORD	50%	
					• RP	50%	

CRB1 = Crumbs homolog 1; CRD = cone-rod dystrophy; EORD = early onset retinal dystrophy; EOSRD = early onset severe retinal dystrophy; LCA = Leber congenital amaurosis; MD = macular dystrophy; RP = retinitis pigmentosa.

∗ MD phenotype when c.498_506del p.(Ile167_Gly169del) is in trans

The c.2506C>A p.(Pro836Thr) variant located in exon 7 was more commonly seen in Black patients (*n* = 4) and Hispanic patients (*n* = 3) from Brazilian cohorts. This variant was strongly correlated with MD; from the 6 homozygous patients with this variant, 5 presented with the MD phenotype, and 1 with RP. However, if present as compound heterozygous then patients presented as either CRD, RP, or MD. Comparably, the c.498_506del p.(Ile167_Gly169del) variant in exon 2, specific to CRB1-A, was identified in 37 individuals from 27 families as compound heterozygous, all presenting with the MD phenotype and, to a lesser extent, CRD (*n* = 5). Interestingly, 3 other individuals with the MD phenotype had mutations specific to CRB1-A, sparing CRB1-B. These included 2 patients with the c.340T>G p.(Cys114Gly) variant in exon 2, present as heterozygous with either the in-frame deletion c.2263_2272del p.(Leu755Alafs∗10) in exon 7 or the c.4009_4015del p.(Ala1337Thrfs∗) in exon 12. Additionally, 1 patient had the c.652+5G>C variant in intron 2, heterozygous with c.2843G>A p.(Cys948Tyr) in exon 9.

**Figure 7:**
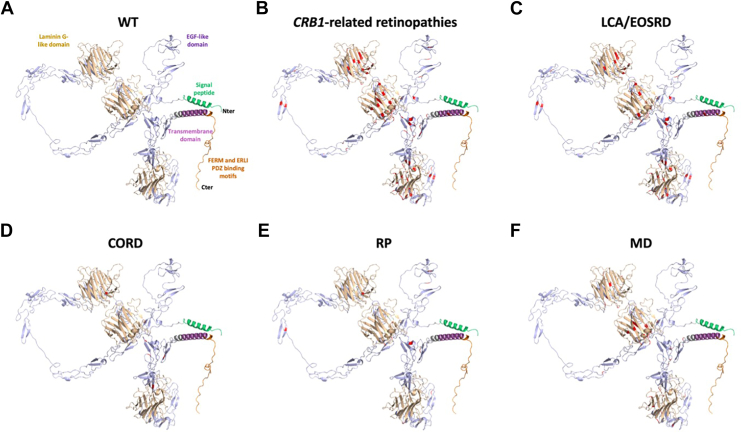
Variants affecting the transmembrane or intracellular domains of CRB1, were linked to Leber congenital amaurosis (LCA)/early onset severe retinal dystrophy (EOSRD). Crumbs homolog 1 protein structure and missense mutation distribution across different *CRB1-*retinopathies highlighting specific protein domains in different phenotypes. **A,** Overall structure of the wild-type (WT) CRB1 protein based on AlphaFold prediction (AF-P82279). Missense variants are depicted in red; signal peptide (N-terminal) in green (amino acid position 1–25); laminin G-like domain in gold; laminin G-like1 (amino acid position 485–670), laminin G-like2 (amino acid position 714–885), laminin G-like3 (amino acid position 960–1137); EGF-like domain in lilac; EGF-like1 (aa position 30–68), EGF-like2 (aa position 70–108), EGF-like3 (aa position 110–146), EGF-like4 (aa position 148–184), EGF-like5 (aa position 186–222), EGF-like6 (aa position 224–260), EGF-like7 (aa position 262–299), EGF-like8 (aa position 301–337), EGF-like9 (aa position 339–395), EGF-like10 (aa position 397–439), EGF-like11 (aa position 441–481), EGF-like12 (aa position 672–708), EGF-like13 (aa position 887–923), EGF-like14 (aa position 924–950), EGF-like15 (aa position 1139–1175), EGF-like16 (aa position 1177–1212), EGF-like17 (aa position 1214–1250), EGF-like18 (aa position 1250–1295), EGF-like19 (aa position 1297–1333). Transmembrane domain in purple (aa position 1348–1348), FERM and ERLI PDZ-binding motifs (C-terminal) depicted in orange (aa position 1369–1406). **B,** Distribution of all identified variants across the CRB1 protein structure in *CRB1*-retinopathies. **C,** Variants associated with LCA/EOSRD, showing a hotspot in the EGF-like14, laminin G-like2, laminin G-like3, and transmembrane/intracellular domains. **D,** Mutations linked to cone-rod dystrophy (CORD) span all protein domains. **E,** Mutations related to retinitis pigmentosa (RP), predominantly located in the EGF-like14, laminin G-like2, laminin G-like3. **F,** Mutations associated with macular dystrophy (MD), primarily observed in the EGF-like4 and laminin G-like2 domains. CRB1 = Crumbs homolog 1; EGF = epidermal growth factor.

Regarding the CRB1 protein structure and mutation distribution across various *CRB1* retinopathies, patients with heterozygous variants in exon 11 and 12, affecting the transmembrane or intracellular domains, were linked to a more severe phenotype (Fig 7). Of the 36 patients who had heterozygous variants affecting the transmembrane or intracellular domains, 24 (66%) were associated with LCA/EOSRD with 12 also having macular atrophy. Furthermore, although there were 2 MD cases (ages 41 and 45 years) reported in patients who carried an exon 12 variant paired with the c.3991C>T p.Arg764His variant in exon 11, they exhibited an atypical extensive nasal macular involvement beyond the optic disc and an unusually rapid 5-year progression of macular atrophy, which is an unusual setting in *CRB1*-MD.^65^ However, when exon 12 variants were paired with heterozygous variants that do not impact isoform CRB1-B, such as c.498_506del p.(Ile167_Gly169del) (*n* = 3) or c.253T>C p.(Cys85Arg) (*n* = 1) in exon 2, a milder MD or CRD phenotype was observed. This suggests that these variants may act as disease modifiers.

Macular atrophy was predominantly associated with the LCA/EOSRD phenotype. Among 50 patients with macular atrophy identified clinically or through retinal imaging, 40 (80%) had LCA/EOSRD, with a mean age of diagnosis of 25 years (±17 years). In contrast, macular atrophy was observed in only 4 patients with RP (8%) and in 3 patients each with MD and CRD (6% per group), with a mean age of diagnosis of 45 years (±9 years). Although the exact timing of initial diagnosis remains unknown for all cases, the age difference suggests that in RP, MD, and CRD, macular atrophy is more likely a feature of advanced disease, whereas in LCA/EOSRD, it may indicate a more inherently severe presentation. The most common genetic variant associated with macular atrophy was c.2843G>A p.(Cys948Tyr), found in 9 patients (18%), followed by c.1831T>C p.(Ser611Pro), which was present in 5 patients (10%). Coats-like vasculopathy was described in 24 patients (16%), of which 13 had LCA/EOSRD. No variant was associated with this presentation. Finally, the c.2506C>A p.(Pro836Thr) variant in exon 7, which was strongly correlated with MD, was also associated in 2 Black patients with ocular hypertension and glaucoma.

## Discussion

This study presents a reappraisal of genotype–phenotype correlations in *CRB1*-retinopathies using a standardized phenotype classification. A total of 389 patients with biallelic *CRB1* variants from 50 international cohorts, including 73 patients from Moorfields Eye Hospital, were analyzed. All patients had biallelic variants affecting CRB1-A, with no cases exhibiting mutations exclusively in CRB1-B, including those in the 1′ and 11′ regions specific to the CRB1-B transcript. Heterozygous variants in exon 11 and 12, affecting the transmembrane/intracellular protein domains of CRB1, were linked to increased disease severity (LCA/EOSRD phenotype ± macular atrophy) as well frameshift variants, nonsense variants, and abnormal splicing variants. Additionally, variants not impacting CRB1-B expression, such as the c.498_506del p.(Ile167_Gly169del) variant, were associated with less severe phenotypes such as MD and CRD.

### CRB1-Retinopathies: Unified Diagnostic Criteria and Genotype–Phenotype Correlations

The initial 10 diagnostic categories observed across different cohorts were reclassified into more defined phenotype groups, confirming that LCA/EOSRD is the most common phenotype among *CRB1*-related retinopathies. The prevalence of LCA/EOSRD was significantly higher than previously reported, accounting for 66% of cases compared with the 17% described in earlier studies.^18^ Although exact frequency of EOSRD alone in *CRB1*-retinopathies has not been previously specified, there is a significant overlap in age of onset and symptoms between LCA and EOSRD, leading to their unification in this analysis and potentially increasing their prevalence.^21^ Retinitis pigmentosa accounted for 16% of patients, lower than the previously reported 37%.^22^^,^^72^ The higher prevalence seen in LCA/EOSRD and lower in RP groups is likely because of the more standardized classification criteria applied in this analysis, which replaced the less specific term “*CRB1*-retinopathy” (originally 22% of cases), and this can potentially enhance genotype–phenotype correlations. Interestingly, 11% of those initially diagnosed with RP were reclassified as LCA/EOSRD, highlighting the overlap of age of onset and clinical features. For instance, a Dutch cohort, which reported 86% of patients with RP, showed a wide range of age of onset, with half experiencing onset in infancy or early childhood;^25^ some individuals in this group may align more closely into the LCA/EOSRD phenotype. Similar observations were noted in a Belgian cohort, suggesting a potential overestimation of the RP phenotype in their study.^17^ Other studies seem to have also classified cases of LCA/EOSRD as RP.^3^^,^^17^^,^^52^^,^^56^ Macular dystrophy was observed in 12% of cases, and CRD in only 5%; exact prevalences have not been previously described across cohorts.

Following the standardized phenotype classification, new genotype–phenotype correlations were identified. The c.2506C>A p.(Pro836Thr) variant was strongly associated with MD if homozygous, but not when compound heterozygous, more prevalent across Black and Hispanic ethnic groups.^73^ This variant was also associated with elevated intraocular pressure and glaucoma.^73^ The c.2506C>A p.(Pro836Thr) variant located in exon 7 affects both CRB1-A and CRB1-B. However, it is predicted to have minimal impact on protein structure, as assessed by Missense3D analysis, which could explain the less severe clinical presentation. Comparably, this study confirmed the already established genotype–phenotype correlation of the in-frame deletion c.498_506del p.(Ile167_Gly169del) associated with a later onset, isolated macular-involving disease with a comparatively better level of visual function than patients with the LCA/EOSRD phenotype.^26^ In contrast, the c.1841G>T p.(Gly614Val) variant was strongly correlated to LCA/EOSRD in both homozygous and compound heterozygous states, and was mainly seen in Asian patients, specifically in Chinese population. Missense3D analysis revealed this variant replaced the buried glycine residue to a buried valine resulting in structural damage to the protein. Overall, these findings highlight the need for accurate clinical phenotyping to support genotype–phenotype correlations.

### CRB1 Gene and In Silico Protein Modeling: Variants in Exons 11 and 12 Are Linked to a More Severe Phenotype

Although a previous report suggested that exons 7 and 9 are the most commonly affected across *CRB1*,^72^ this study found that variants in exons 6 were also frequently noted, and were predominantly associated with LCA/EOSRD and RP. Mutations in exon 2, particularly c.498_506del p.(Ile167_Gly169del), were linked to MD and CRD. Targeting exons 2, 6, 7, and 9 through cluster regularly interspaced short palindromic repeats (CRISPR)-Cas–mediated base editing or focused diagnostic panels could capture the majority of *CRB1* variants, improving therapeutic strategies while enabling cost-effective genetic testing that would resolve around 62% of suspected cases.^12^

Frameshift, nonsense, and splice variants were strongly associated with LCA/EOSRD in this study. Splice and frameshift variants typically introduce premature stop codons, leading to truncated proteins or a complete absence of protein expression, functioning similarly to null variants because of their significant impact on protein function. Additionally, heterozygous variants in exons 11 and 12, regardless of the type, were linked to LCA/EOSRD ± macular atrophy, as they affect the transmembrane or intracellular domains of CRB1. Previously, Mairot et al^7^ reported that the c.4073T>A p.(Ile1358Asn) variant, located in exon 12, is predicted to destabilize the transmembrane segment, replacing a hydrophobic isoleucine with a polar asparagine, potentially leading to improper membrane insertion or faulty multimerization. Similarly, variants in exon 12 such as c.4214del p.(Leu1405Argfs∗82) and c.4219T>A p.(Ter1407Lysext∗111) likely result in an extension of the intracellular protein segment, which interacts with partners like PALS1, crucial for stabilizing the CRB complex, and Moesin, which binds to the FERM-binding domain.^7^ However, patients with a variant in exon 12 and either c.498_506del p.(Ile167_Gly169del) or c.253T>C p.(Cys85Arg) variants in exon 2 in *trans,* manifested a milder phenotype, with either MD or CRD. No other correlation between the location of *CRB1* mutations and specific protein domains across different phenotypes was found. This highlights the complexity of *CRB1*-retinopathies and suggests that factors beyond mutation location within the protein structure may contribute to the phenotypic variability observed.

One potential contributing factor to phenotypic variability is the dysregulation of epigenetic mechanisms as a pathological process. Owen et al^14^ demonstrated altered epigenetic regulation in the *crb2a* (*crb2a* ^*m289/m289*^, termed *crb2a*^*–/–*^) zebrafish model, affecting gene expression, chromosome condensation, methylation, and visual transduction.^14^ The loss of *crb2a* disrupted the gene regulatory network controlling cell cycle progression, significantly altering the methylation signature of the developing retina. This was accompanied by increased expression of VSX2 and PAX6, indicating an enrichment of retinal progenitor cell population with excessive proliferation and impaired cell fate progression. Notably, the absence of photoreceptors was not attributed to increased apoptosis but rather to a prolonged proliferative state, leading to a thickened, disorganized retinal structure with reduced cell cycle exit.^14^ Future studies comparing epigenetic signatures between phenotypes may help elucidate the phenotypic variability observed in *CRB1*-retinopathies.

### CRB1 Isoforms: Can CRB1-A and CRB1-B Influence the Phenotype?

In recent years, studies on retinal cell expression of CRB1 isoforms have helped improve genotype–phenotype correlations, especially for the MD phenotype. This study revealed that all patients had disease-causing variants affecting CRB1-A, with none exclusively affecting CRB1-B, indicating that CRB1-A must be affected to develop the disease. Crumbs homolog 1-A is primarily expressed in Müller cells and plays a vital role in retinal development, whereas CRB1-B, expressed in photoreceptors, helps maintain long-term retinal integrity in adult retinas.^7^ Several variants identified in this study, specific to CRB1-A while sparing CRB1-B, were associated with the MD phenotype. For example, the in-frame deletion c.498_506del p.(Ile167_Gly169del) in exon 2 was predominantly linked to MD, with most patients carrying this variant exhibiting the condition, suggesting it is a predictive disease marker. This variant destabilizes the local folding and orientation of 2 epidermal growth factor-like modules, compromising the structural organization of the CRB1 protein, yet sparing CRB1-B function. In addition, 2 patients with MD carried the c.340T>G p.(Cys114Gly) variant in exon 2, present as compound heterozygous with either the in-frame deletion c.2263_2272del p.(Leu755Alafs∗10) in exon 7 or the c.4009_4015del p.(Ala1337Thrfs∗2) in exon 12. Finally, another patient with MD had the c.652+5G>C variant in intron 2, heterozygous with c.2843G>A p.(Cys948Tyr) in exon 9. If CRB1-B is spared, it could potentially ameliorate the disease by still providing function and certain level of integrity in the adult retina. These findings may explain the less severe impact on visual acuity, a localized macular involvement, preserved retinal lamination seen on spectral-domain OCT, and a later age of onset (median of 18 years) observed in this study.^18^^,^^22^

It is important to highlight that 4 patients carrying the c.498_506del p.(Ile167_Gly169del) variant as compound heterozygous with an allele linked to disease severity presented as CRD. Three of these patients had a nonsense variant, and 1 had a splice-site variant, both leading to truncated proteins or complete loss of expression, suggesting the role of the second affected allele in contributing to increased disease severity and revealing that the c.498_506del p.(Ile167_Gly169del) variant can also manifest as CRD.

Macular dystrophy was not exclusively seen because of preservation of CRB1-B on one allele, as it also appeared in patients with variants affecting CRB1-B in both alleles. An example of this are the missense mutations c.1562C>T p.(Ala521Val), c.2234C>T p.(Thr745Met), and c.2506C>A p.(Pro836Thr). Missense3D analysis predicts these variants to have minimal structural impact on the protein, which may contribute to the isolated macular-involving presentation as compared with patients with the LCA/EOSRD phenotype. The c.1562C>T p.(Ala521Val) mutation, despite involving a change between alanine and valine, is predicted to be disease-causing, suggesting a potentially hypomorphic effect. However, the precise mechanism by which these variants lead to MD remains unclear, largely because of the absence of corresponding mouse models or MD-CRB1 induced pluripotent stem cell lines for further investigation.

### CRB1-Retinopathies Associations: Coats-like Vasculopathy and “Pseudo-coloboma”

Coats-like vasculopathy was found in 24 (16%) cases, of which 13 had LCA/EOSRD with no variant associated with this presentation. Several disease mechanisms and pathways have been proposed to explain why Coats-like vasculopathy is seen more frequently in *CRB1-*retinopathies compared with other inherited retinal diseases caused by genes such as *RHO, USH2A*, and *MYO7A*.^74^ First, it is thought that Coats-like vasculopathy is secondary to a breakdown in the normal blood–retinal barrier mediated by loss of normal zonula occludens function and therefore immune exposure to retinal antigen.^3^ Second, as Müller cells function as regulators of the tightness of blood–retinal barrier, dysfunction of these cells could lead to vascular abnormalities seen in *CRB1* patients.^75^ Third, Son et al^76^ reported that CRB control retinal angiogenesis by regulating neuroglial VEGF-A and matrix metalloproteinase-3 expression, demonstrating an important role of CRB1 in providing neurotrophic support through normal layered vascular network development and maintenance.^76^ Finally, Coats-like vasculopathy in *CRB1* could be associated with dysregulation of the Wnt signaling pathway, which plays a significant role in vascular morphogenesis including development of structured layers of vasculature in retina.^77^ Loss of function variants in Wnt signaling components are associated to cause Norrie disease, and familial exudative vitreoretinopathy with defect ocular vasculature.^77^ Weed et al^78^ showed that *CRB1*-LCA patient-derived retinal progenitor cells have altered expression of several Wnt signaling pathway components including *AXIN2*, *LEF1*, *TCF7*, and *WNT3a* between day 14 and 21. Further Wnt signaling pathways in animal models and other *CRB1* patient-derived (retinal progenitor cell) lines should be carried out to confirm this.

Finally, the presence of macular atrophy was strongly associated with the LCA/EOSRD phenotype and more commonly presented with the c.2843G>A p.(Cys948Tyr) variant, aligning with previous reports that macular atrophy was associated with LCA phenotype.^3^^,^^17^ This is particularly important, because LCA “pseudo-coloboma” cases have been mostly associated with variants in *CRX, AIPL1, NMNAT1, LCA5,* and *IDH3A*. Therefore*, CRB1-*LCA should also be considered as a differential diagnosis in an LCA patient with “pseudo-coloboma.”

### Future Directions

As research on treatment strategies advances, studying various *CRB1* induced pluripotent stem cell models with different phenotypes will be crucial for understanding the disease mechanisms and identifying pathways that can be targeted with specific drugs or therapies aimed at specific mutations. In addition, natural history studies, particularly prospective ones, are crucial for understanding the time course and pattern of disease progression, determining the therapeutic window of opportunity, patient eligibility criteria, and reliable clinical trial outcome metrics.^79^ They are an essential part of the drug development process, as they inform clinical trial design, duration and frequency of data collection, and possible outcome measures as potential trial points. Recently, several retrospective and prospective studies have started to evaluate the optimal therapeutic window for *CRB1*-retinopathies.^80^ For patients with *CRB1*-LCA/EOSRD, therapeutic intervention for EOSRD will likely need to occur within the first decade of life because of early functional and structural involvement of the macula. Patients with LCA would benefit from intervention within the first year of life. In contrast, patients with *CRB1*-RP, CRD, and MD have a more extended therapeutic window spanning the first 3 decades of life.^47^ A prospective 2-year deep phenotyping study of patients with *CRB1* is now underway and baseline measurements have been completed. Aside from molecular confirmation, the inclusion criteria involve having a visual acuity of 20/200 (or 1.0 LogMAR) or better in the better-seeing eye. The chosen cut-off vision score was intended to ensure that the included patients can perform tests requiring stability fixation, including psychophysical assessments. This will enable a focus on characterizing the early signs of disease, determining disease progression, providing insight into prognosis, and establishing a set of age-appropriate tests suitable for clinical trial outcome metrics.

### Limitations

This study was a retrospective observational analysis rather than a systematic review, and has several inherent limitations. Firstly, the methodology involved the selection of *CRB1* biallelic patients for whom sufficient phenotypic and clinical data were available for genotype–phenotype association analysis, which by necessity excluded individuals not fully characterized at the ocular level, even if complete genetic information existed. This approach introduces selection bias, which might alter the overall prevalence of certain *CRB1* mutations and their clinical associations. As a narrative review relying on data from 50 international cohorts, the study is further susceptible to publication bias and heterogeneity in clinical and genetic data curation across different centers.

Although we undertook a detailed re-evaluation and standardized phenotypic classification of patients based on clinical data, age of onset, vision, and ERG, performed by 2 ophthalmologists specializing in genetics, we acknowledge the absence of a process for central re-evaluation, harmonization, or independent adjudication of phenotypic labels by expert panels. This omission is critical, as it introduces a potential for phenotypic misclassification, which could affect the validity of the genotype–phenotype correlations reported. Full clinical descriptions and detailed genetic associations for all patients were challenging because of inconsistencies in the clinical data reported across various cohorts. Finally, a uniform description of retinal architecture was not performed because of the low number of individuals with standardized OCT measurements and a general lack of detailed individual characterization across all included cohorts.

### Conclusion

A reappraisal of the genotype–phenotype correlations in *CRB1*-related retinopathies was conducted based on the available literature. All patients included had biallelic variants affecting CRB1-A, with no cases exhibiting mutations exclusively in CRB1-B. Patients with mutations involving exons 11 and 12, affecting the transmembrane or intracellular domains of CRB1, were linked to a severe disease, presenting with LCA/EOSRD plus or minus macular atrophy. Gaining a fundamental understanding of the *CRB1* gene and protein function is essential for addressing the heterogeneity of *CRB1*-retinopathies. Having clear diagnostic guidelines and global consensus of phenotypic classification of patients with *CRB1*-retinopathies is essential as it not only enhances our ability to advise patients but also facilitates the development of targeted therapies and the design of more effective clinical trials. There is a need for more *CRB1* natural history studies to provide additional evidence and achieve a global consensus of diagnostic criteria.

## Figures and Tables

**Supplementary figure:**
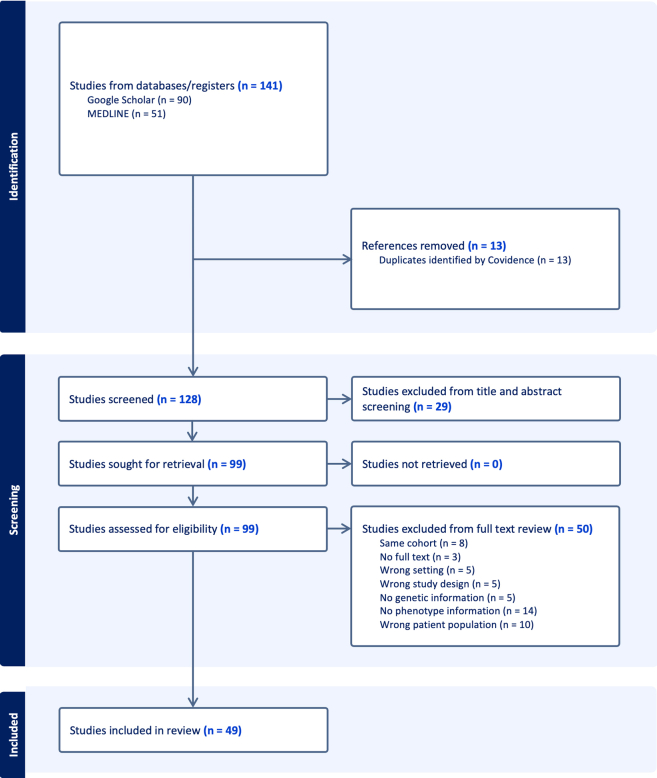

